# Prevalence of Noise-Induced Hearing Loss Among Construction Workers in Puducherry, India

**DOI:** 10.7759/cureus.72804

**Published:** 2024-10-31

**Authors:** Anbukarasi Ramalingam, Prem Davis, Pournima Ganesan, Prabu Velayutham

**Affiliations:** 1 Otorhinolaryngology, Head and Neck Surgery, Panimalar Medical College Hospital and Research Institute, Chennai, IND; 2 Health Centre, Central University of Tamil Nadu, Thiruvarur, IND; 3 Otorhinolaryngology, Head and Neck Surgery, Sri Venkateshwaraa Medical College Hospital and Research Centre, Puducherry, IND; 4 Otorhinolaryngology, Head and Neck Surgery, Army Medical Corps, Leh, IND

**Keywords:** construction, hearing loss, impairment, noise, workers

## Abstract

Introduction

Noise-induced hearing loss (NIHL) is one of the emerging health problems in today's world. NIHL results from multifactorial damage to auditory structures due to exposure to loud sounds from occupational, environmental, or recreational causes. The World Health Organisation (WHO) indicates that workplace NIHL is second to accidental harm for years of healthy life lost. It causes significant impairment in patients' daily activities and quality of life.

Aim

This study aimed to find the prevalence of NIHL among construction site workers in Puducherry, a southern state of India, and to find the association between the degree of hearing loss and various demographic factors.

Methods

This cross-sectional study was conducted among construction workers in a tertiary care center in Puducherry. The study was conducted for 18 months, from January 2021 to June 2022. After the initial assessment, all the workers were subjected to pure tone audiometry, and findings were recorded.

Results

A total of 500 construction site workers participated in the study. The prevalence of the NIHL was found to be 13.2%. The study also showed that the majority of the patients (6.6%) had a mild-degree hearing loss. The study also showed a significant association exist between age (p = 0.002), gender (p = 0.001), work experience (p < 0.001), and the number of hours of work per day (p < 0.001) with the degree of hearing loss.

Conclusion

The prevalence of NIHL is growing day by day, so proper preventive measures should be taken by enhancing knowledge of the disease, adopting protective tactics, and creating therapies to prevent the disease.

## Introduction

Noise is a pervasive industrial contaminant, affecting all industries and resulting in significant hearing loss globally. Occupational hearing loss includes acoustic traumatic injury and noise-induced hearing loss (NIHL) [1]. The NIHL results from multifactorial damage to auditory structures due to exposure to loud sounds from occupational, environmental, or recreational causes. Occupational NIHL may occur with sustained exposure to noise levels of 85 dB or higher for eight hours per day or 40 hours per week [2]. While earplugs were patented in 1864, hearing protection devices are referenced in ancient Greek mythology [3].

NIHL was officially recognized as a medical ailment in the United States of America during the Industrial Revolution, initially termed "boilermaker's disease" to denote the auditory impairment experienced by workers constructing engines for transportation and industry [4]. Not only the United States but developing countries such as India were also affected by NIHL. NIHL is a significant public health concern, as increasing life expectancy and industrialization will considerably contribute to the worldwide disability burden. The World Health Organization (WHO) indicates that workplace NIHL is second to accidental harm for years of healthy life lost [5].

Studies have shown that in most countries, excessive noise production is one of the most significant compensatable occupational hazards to most workers and people. Globally, 16% of adult debilitating hearing loss is ascribed to occupational noise, with variations between 7% and 21% across different subregions. The projected economic impact of noise on developed nations varies between 0.2% and 2% of GDP, contributing to over one-third of hearing impairments [6]. Moreover, it indirectly affects the quality of life of the patients. Studies have shown that exposure to occupation noise was higher among people in developing countries than in developed countries [7].

The duration and severity of NIHL are contingent upon the degree and site of cellular damage, which correlates with the strength and duration of the auditory input [8]. The mammalian auditory sensory epithelium, known as the organ of Corti, lacks the capacity for spontaneous regeneration following the loss of sensory cells; thus, noise-induced degeneration of hair cells or neural structures can lead to irreversible hearing loss, especially with repeated exposure [9]. Only a few studies have been done to describe the prevalence and risk factors of NIHL among construction workers in India. Thus, this study was planned to assess the prevalence of NIHL among construction workers in India.

## Materials and methods

Study design

This cross-sectional study was conducted among construction workers in a tertiary care center (Sri Venkateshwaraa Medical College Hospital and Research Centre) in Puducherry, India. The study was conducted for 18 months, from January 2021 to June 2022.

Sampling

All the consultation workers who visited the hospital during the study period were included. The patients were selected using the convenient sampling method. As such, a total of 500 construction workers who visited the hospital were included in the study.

Inclusion Criteria

The construction workers over 18 years and below 60 who visited the hospital during the study were included.

Exclusion Criteria

Patients who are less than 18 years and more than 60 years old, patients with ear and nasal complaints who had previous ear and nasal surgeries, and patients diagnosed with uncontrolled diabetes, cancer, or any psychological disorder were excluded from the study. Patients previously diagnosed with hearing loss were also excluded from the study.

Data collection

After obtaining approval from the Institute Ethics Committee and informed written consent, construction site workers who visited the hospital with conditions other than nose and ear were included in the study. Detailed histories were taken in all patients, followed by systemic and ENT examinations. The ear examination was done initially under a bull's eye lamp, followed by otoscopic examination. Otoendoscopic examination was done in needed cases only. Then, a nose and throat examination was done. All the patients were then subjected to pure tone audiometry. Audiometric testing was performed in a soundproof environment using a calibrated digital audiometer (Viola Inventis, India). Hearing thresholds were measured for each ear at frequencies ranging from 250 Hz to 8 kHz, with a threshold over 25 dB classified as hearing loss at any of these frequencies. Patient information was recorded on the clinical PROforma. The workers with a hearing threshold of less than 25 decibels (dB) were taken as normal hearing, those with a hearing threshold between 26 dB and 40 dB as mild hearing loss, those with 41 to 55 dB as moderate hearing loss, those with between 56 and 70 as moderately severe loss, those between 71 and 90 as severe, and those with more than 91 as profound hearing loss [10].

Data analysis

Data entry was conducted using the EpiData software (EpiData Association, Denmark), and analysis was performed with the IBM SPSS Statistics for Windows, Version 25.0 (released 2017, IBM Corp., Armonk, NY). The chi-square test and the Fisher's exact test were used to find the statistically significant association between the variables. 

Ethical approval

The study protocol received approval from the Internal Human Ethics Committee of Sri Venkateshwaraa Medical College Hospital and Research Centre, Ariyur, Puducherry, under reference number SVMCH/OEC/2019-Nov/27.

## Results

A total of 500 construction site workers participated in the study. About 376 (75.2%) of the workers were between 18 and 30 years, followed by 90 (18%) of the patients between 31 and 40 years, 31 (6.2%) between 41 and 50 years, and only three (0.6%) were between 51 and 60 years of age. Male workers contribute to 320 (64%) of the total study population. In the study, more than half of the workers, 298 (59.6%), had work experience between one and 50 years, followed by 160 (32%) who had six to 10 years, and 23 (4.6%) had 11 to 15 years of work experience. About 11 (2.2%) had 21 to 30 years and eight (1.6%) had 16 to 20 years of work experience or exposure in the same field. A total of 290 (58%) workers work eight to 10 hours per day, followed by 150 (30%) workers who work for more than 10 hours per day, as shown in Table 1.

**Table 1:** Sociodemographic details of the study participants (N = 500)

Variable	Frequency (N)	Percentage (%)
Age (in years)		
18-30	376	75.2
31-40	90	18
41-50	31	6.2
51-60	3	0.6
Gender		
Male	320	64
Female	180	36
Work experience (in years)		
1-5	298	59.6
6-10	160	32
11-15	23	4.6
16-20	8	1.6
21-30	11	2.2
No hours of work per day		
1-5	10	2
5-8	50	10
8-10	290	58
>10	150	30

**Figure 1:**
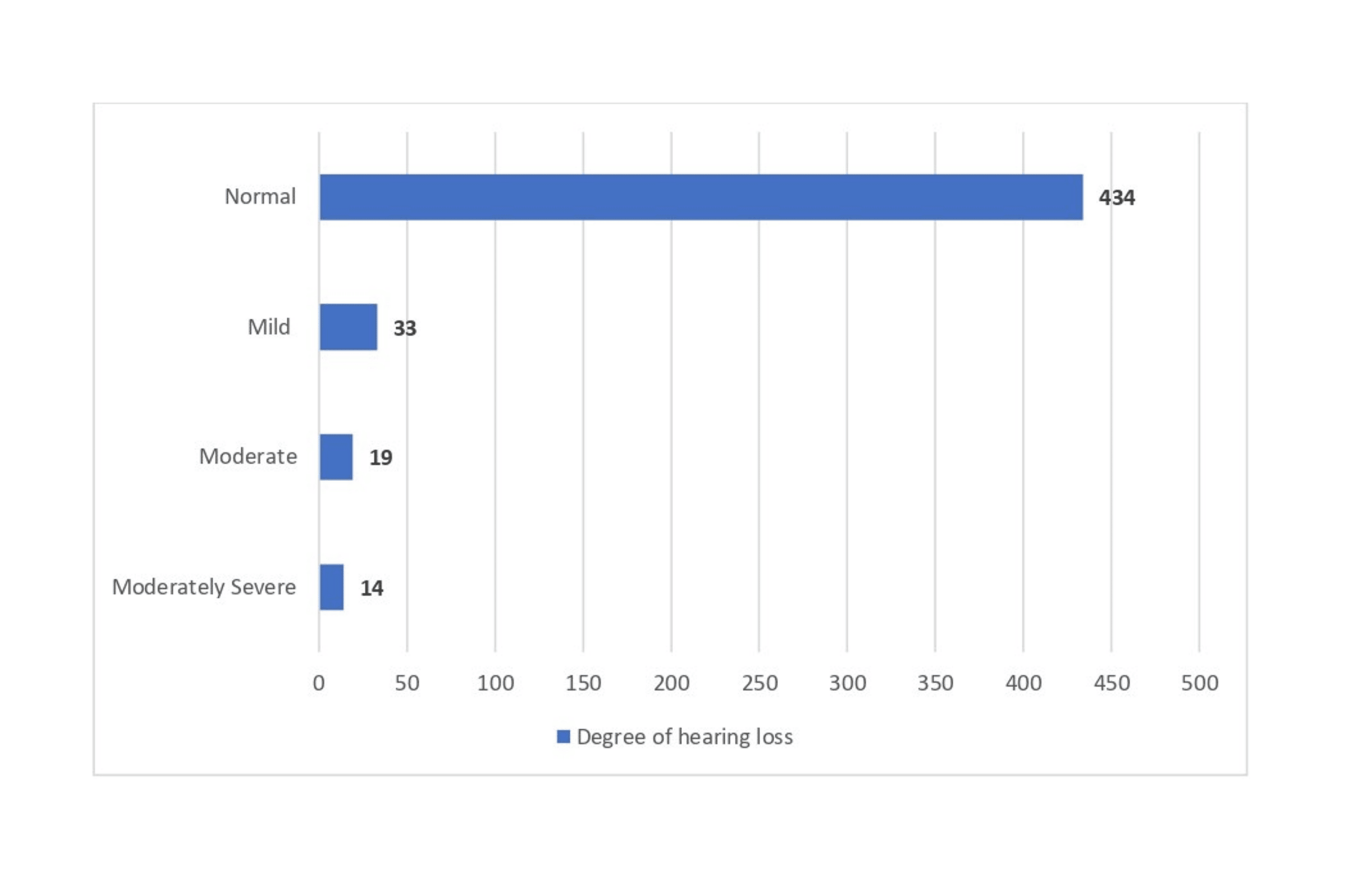
Distribution of the consultation site workers based on the degree of hearing loss (N = 500)

The incidence of NIHL among the construction workers was 66 (13.2%). The study also showed that about 33 (6.6%) of the workers had mild hearing loss, followed by 19 (3.8%) of the patients with a moderate degree and 14 (2.8%) with moderately severe hearing loss, as shown in Figure 1.

In finding the association between the various sociodemographic variables and the degree of hearing loss in the younger age group, it was found that about 24 (6.4%) of the patients had a mild degree of hearing loss. It was also noted in the 41 to 50 years, whereas in the middle age group, 31 to 40 years, about seven (7.8%) had moderate hearing loss. The study also showed that the majority of the male patients had moderate (14, 4.4%) and moderately severe (13, 4.1%) hearing loss, whereas females (23, 12.8%) had mild degrees of hearing loss. The study also showed that workers with eight to 10 years of work experience had moderately severe hearing loss (14, 4.8%). About 25 (16.7%) workers who work more than 10 hours daily have severe hearing loss. The study also showed a significant association between age (p = 0.002), gender (p = 0.001), work experience (p < 0.001), and the number of hours of work per day (p < 0.001), as shown in Table 2.

**Table 2:** Association between the sociodemographic variables and the degree of hearing loss among the construction site workers. A p-value less than 0.05 is taken as significant (p < 0.05*, p < 0.001**).

Variable	Normal N (%)	Mild N (%)	Moderate N (%)	Moderately severe N (%)	Total N (%)	Chi-square value	p-value
Age (in years)							
18-30	334 (88.8)	24 (6.4)	10 (2.6)	8 (2.2)	376 (100)	25.32	0.002*
31-40	75(83.3)	4 (4.4)	7 (7.8)	4 (4.4)	90 (100)		
41-50	24 (80)	4 (13.3)	1 (3.3)	1 (3.3)	30 (100)		
51-60	1 (25)	1 (25)	1 (25)	1 (25)	4 (100)		
Gender							
Male	283 (88.4)	10 (3.1)	14 (4.4)	13 (4.1)	320 (100)	22.37	0.001*
Female	151 (83.9)	23 (12.8)	5 (2.8)	1 (0.5)	180 (100)		
Work experience (in years)							
1-5	269 (90.3)	19 (6.4)	8(2.7)	2 (0.7)	298 (100)	64.71	<0.001**
6-10	142 (88.8)	5 (3.1)	5 (3.1)	8(5)	160(100)		
11-15	13 (56.5)	6 (26.1)	3 (13.1)	1 (0.2)	23(100)		
16-20	3 (0.6)	1 (0.2)	2 (0.4)	2 (4.3)	8(100)		
21-30	7 (63.6)	2 (18.2)	1 (9.1)	1 (9.1)	11(100)		
No. of hours of work per day (in hours)							
1-5	6 (60)	2 (20)	1 (10)	1 (10)	10(100)	65.83	<0.001**
5-8	47 (94)	1 (2)	1 (2)	1 (2)	50 (100)		
8-10	270 (93.1)	5 (1.7)	14 (4.8)	1 (0.3)	290 (100)		
>10	111 (74)	25 (16.7)	3 (2)	11 (7.3)	150 (100)		

## Discussion

The noise originates from human activity, particularly urbanization and expanding transport and industries. The term noise originates from the Latin word "nausea," signifying an impetuous, unwelcome, unpleasant, or unexpectedly loud sound [11]. The correlation between occupational noise exposure and hearing loss is well-established. Despite implementing different preventive measures to mitigate noise impact, it continues to be one of the most common occupational health hazards. It has numerous detrimental effects, including hypertension, anxiety, disorientation, cephalalgia, diminished performance, sleep disturbances, irritability, stress, tinnitus, and, ultimately, permanent, irreversible sensorineural hearing loss [12].

Noise is reaching critically frightening levels and is becoming damaging in all areas of life, particularly for industrial workers. In our study, all workers, whether in the office, as machinery operators, or as assistants, who were exposed to noise developed hearing impairments [13]. Prolonged exposure to noise levels over 85 dB for more than eight hours daily induces shearing forces on the stereocilia of hair cells along the cochlea's basilar membrane, resulting in the degradation of the fragile structures within the inner ear [1].

Occupational noise-induced hearing loss is a significant issue for workers in industries such as military forces, aviation, maritime, heavy machinery, construction sites, and weaponry, where there is persistent exposure to loud environments [14]. Noise stress is recognized to induce a 4 kHz notch, sometimes called an Aviator's notch. The phenomenon of permanent hearing loss characterized by a 4 kHz notch from noise-induced damage arises from the heightened sensitivity of human hearing in the 1-5 kHz range, the acoustic reflex's attenuation of loud sounds below 2 kHz, and the nonlinear functioning of the middle ear at elevated intensities [15]. The maximum allowable occupational noise limit established by the International Standards Organisation is 85-90 dBA for an eight-hour workday. The model guidelines under the Indian Factories Act of 1948 establish a permissible noise limit of 90 dBA for an eight-hour exposure over six days per week in India [16]. Most workers in skilled or semi-skilled industrial roles need to be more literate or semi-illiterate. They need more awareness of noise regulations and the detrimental impact of noise on their performance and health [17].

In our study, the prevalence of NIHL among construction workers was 13.2%. The study by Ranga RK et al. [16] showed that the prevalence of noise-induced hearing loss was found to be 12.8%, and the study also showed that workers belonging to 36 to 40 years of age of found to be more commonly affected with NIHL than the other age groups patients. The study by Edward M et al. [18] showed that among the 111 workers who were exposed to the noise, about 51.85% of the patients had NIHL, which was much higher than our study results. The same study also showed that the majority of patients (56.1%) had a mild degree of hearing loss, followed by 38.6% of the patients with moderate hearing loss and 5.3% with moderate to severe hearing loss, which was similar to our study results. Ahmed et al. [19] determined that almost 89% of workers were exposed daily to levels exceeding the permitted limit of 85 dB and 45% had never utilized hearing protection devices. Approximately 58% of the employees reported experiencing moderate to high noise irritation. In our study, a significant association was seen between gender and the degree of hearing loss. Our study showed that male patients had more hearing loss than female patients. The study by Nelson DI et al. [4] also showed that exposure to occupational noise was found to be more common among male patients than female patients, and it was found to be more common in the regions of developing countries. This occupational noise was one of the significant causes of adult-onset hearing loss.

Our study also showed a significant association between the number of years of work and the number of work hours per day. Moreover, the study by Edward M et al. [18] showed similar results to our study, where the patients with increased total exposure to the work were associated with a higher degree of hearing loss. In a study by Musiba et al. [20], the proportion of NIHL was increased with the total years of exposure to the noise environment. The study also showed that underground mines were more commonly affected (71%) than open-pit miners (28%). The study also showed that the highest proportion of miners with NIHL was found in workers between 20 and 29 years of age. Wallhagen et al. [21] reported that the long-term effects of noise exposure in young people are exacerbated by greater NIHL exposure during early childhood. The mobility of the middle ear diminishes with age. Therefore, a less effective transfer across the middle ear may also reduce susceptibility to NIHL.

Limitation

The study was done on a single center, so it may not be able to represent the whole population. Hence, if it is done as a multi-center study, it will help to represent the whole population.

## Conclusions

NIHL is one of the emerging health problems in today's world, significantly impacting health and day-to-day activities. The prevalence of NIHL among construction site workers was 13.8% in our study. NIHL affects the people exposed to it and the general public due to the rapid growth of urbanization. Nonetheless, NIHL is predominantly preventable if suitable precautions, such as hearing protection, are implemented. Consequently, instituting steps to identify and reduce causative variables, enhancing knowledge of the disease, adopting protective tactics, and creating therapies to safeguard against or alleviate harm from noise exposure will prevent this prevalent ailment.
